# Longitudinal associations of fast foods, red and processed meat, alcohol and sugar-sweetened drinks with quality of life and symptoms in colorectal cancer survivors up to 24 months post-treatment

**DOI:** 10.1017/S0007114522003051

**Published:** 2023-07-14

**Authors:** Marlou-Floor Kenkhuis, Floortje Mols, Eline H. van Roekel, José J. L. Breedveld-Peters, Stéphanie Breukink, Maryska Janssen-Heijnen, Eric Keulen, Fränzel J. van Duijnhoven, Matty P. Weijenberg, Martijn Bours

**Affiliations:** 1 Department of Epidemiology, GROW School for Oncology and Reproduction, Maastricht University, Maastricht, the Netherlands; 2 Department of Medical and Clinical Psychology, Tilburg University, Tilburg, the Netherlands; 3 Department of Surgery, GROW School for Oncology and Reproduction, NUTRIM School of Nutrition and Translational Research in Metabolism, Maastricht University Medical Centre+, Maastricht, the Netherlands; 4 Department of Clinical Epidemiology, Viecuri Medical Center, Venlo, the Netherlands; 5 Department of Internal Medicine and Gastroenterology, Zuyderland Medical Centre, Sittard-Geleen, the Netherlands; 6 Division of Human Nutrition and Health, Wageningen University & Research, Wageningen, the Netherlands

**Keywords:** Colorectal cancer survivorship, Lifestyle recommendations, Diet, Health-related quality of life, Fatigue, Chemotherapy-induced peripheral neuropathy

## Abstract

Unhealthy dietary habits can contribute to the development of colorectal cancer (CRC). Such habits may also be associated with post-treatment symptoms experienced by CRC survivors. Therefore, we aimed to assess longitudinal associations of post-treatment unhealthy dietary habits, i.e. intake of ultra-processed foods (UPF), red and processed meat, alcohol and sugar-sweetened drinks, with health-related quality of life (HRQoL), fatigue and chemotherapy-induced peripheral neuropathy (CIPN) in CRC survivors from 6 weeks up to 24 months post-treatment. In a prospective cohort among stage I-III CRC survivors (*n* 396), five repeated home visits from diagnosis up to 24 months post-treatment were executed. Dietary intake was measured by 7-d dietary records to quantify consumption of UPF, red and processed meat, alcohol and sugar-sweetened drinks. HRQoL, fatigue and CIPN were measured by validated questionnaires. We applied confounder-adjusted linear mixed models to analyse longitudinal associations from 6 weeks until 24 months post-treatment. We applied a *post hoc* time-lag analysis for alcohol to explore the directionality. Results showed that higher post-treatment intake of UPF and sugar-sweetened drinks was longitudinally associated with worsened HRQoL and more fatigue, while higher intake of UPF and processed meat was associated with increased CIPN symptoms. In contrast, post-treatment increases in alcohol intake were longitudinally associated with better HRQoL and less fatigue; however, time-lag analysis attenuated these associations. In conclusion, unhealthy dietary habits are longitudinally associated with lower HRQoL and more symptoms, except for alcohol. Results from time-lag analysis suggest no biological effect of alcohol; hence, the longitudinal association for alcohol should be interpreted with caution.

Colorectal cancer (CRC) survival rates continue to rise due to screening, improved treatment and an aging population^(1–3)^. Consequently, an increasing amount of people are living with and beyond CRC. However, a CRC diagnosis and subsequent treatments can lead to considerable detriments in physical and mental health^(4–6)^. Two common symptoms affecting health-related quality of life (HRQoL) of CRC survivors include fatigue and chemotherapy-induced peripheral neuropathy (CIPN)^(7–9)^. The increasing number of CRC survivors highlights the importance for identifying ways to enhance their HRQoL and alleviate symptoms of fatigue and CIPN. The diet of CRC survivors is a potential candidate for alleviating symptoms since unhealthy dietary habits could have contributed to the development of CRC and may also affect HRQoL and symptoms after diagnosis and treatment.

The majority of the 2018 World Cancer Research Fund/American Institute for Cancer Research (WCRF/AICR) lifestyle recommendations on cancer prevention include specific recommendations on diet^(10)^. These dietary recommendations can be split up in those that promote healthy dietary habits (e.g. increase intake of fruit and vegetable and fibre) and those that warn against unhealthy habits. In this article, we focus on the latter. Specifically, WCRF/AICR recommends to limit consumption of fast foods and other processed foods high in fat, starches or sugar; to eat no more than three portions of red meat a week and eat little, if any, processed meat; to not drink alcohol and to limit consumption of sugar-sweetened drinks.

The WCRF/AICR dietary recommendations for cancer prevention are predominantly based on evidence from numerous aetiological studies^(10)^. The few studies that have investigated the importance of these recommendations in cancer survivors focus mostly on recurrence and survival^(11)^. Currently, little evidence is available regarding these dietary recommendations and how this is related to HRQoL, fatigue and CIPN in CRC survivors. Among CRC patients 2 to 10 years post-diagnosis, we previously observed cross-sectional associations of higher intake of energy-dense foods with worse physical functioning and more fatigue, but no associations were observed for sugar-sweetened drinks, and red and processed meat^(12)^. No associations with CIPN were found. Strikingly, non-alcoholic drinkers had statistically significantly lower levels of physical, role and social functioning, and higher levels of fatigue compared with moderate alcohol drinkers^(12)^. Similar associations for alcohol intake were observed by Grimmett *et al.* ^(13)^

There is a need for more longitudinal research on the association between post-diagnosis dietary intake and HRQoL, fatigue and CIPN. We aim to examine longitudinal associations of the WRCF/AICR dietary recommendations focusing on limiting unhealthy habits, including fast foods, red and processed meat, alcohol and sugar-sweetened drinks, with HRQoL, fatigue and CIPN in CRC survivors from 6 weeks up to 24 months post-treatment.

## Methods

### Study design and population

**Fig. 1.**
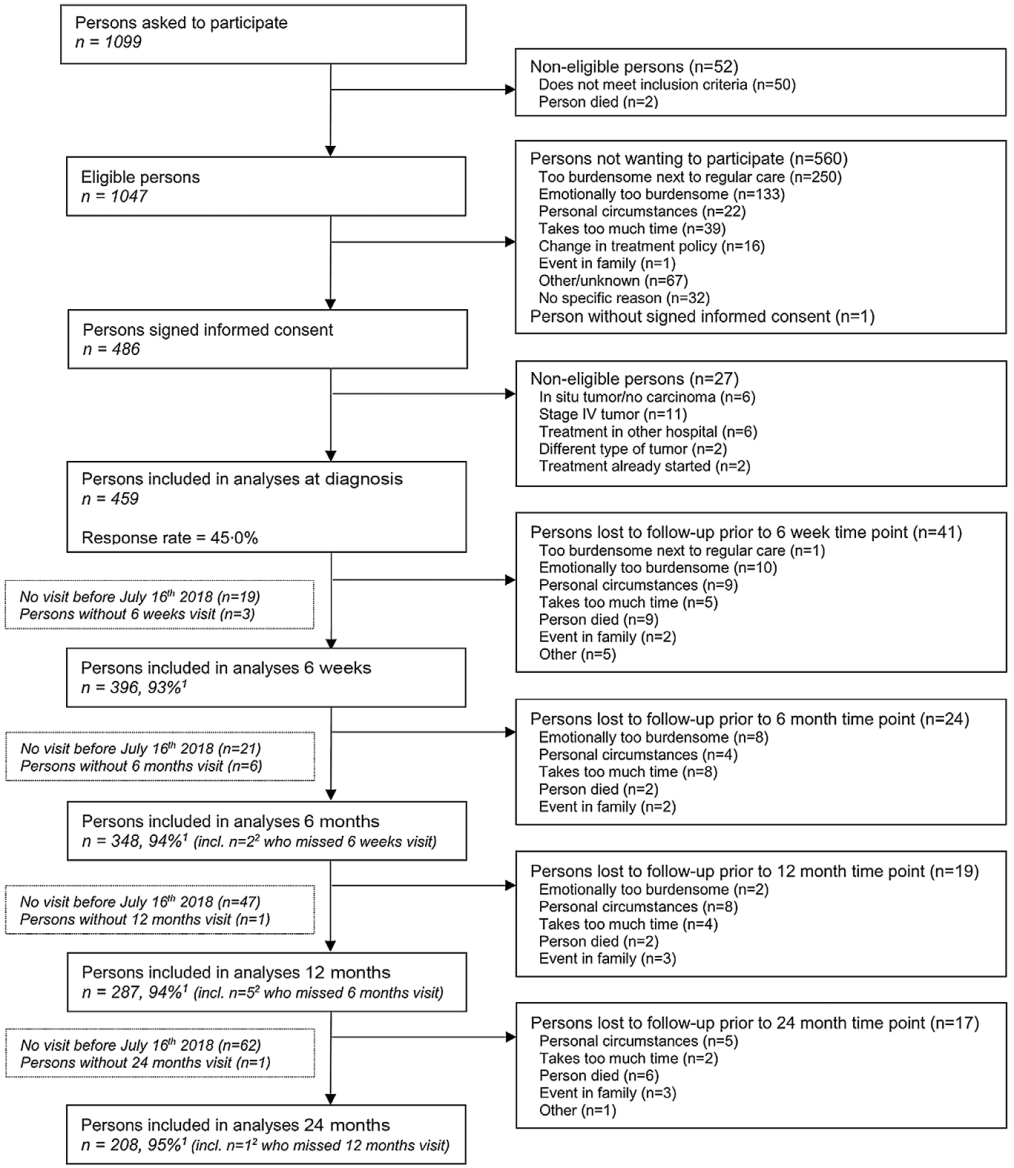
Flow diagram of inclusion of individuals within the EnCoRe study and included in the analyses presented in this paper. Data of home visits performed before 16 July 2018 were included in the analyses. ^1^Response rate post-treatment = (persons included)/(persons included + persons lost to follow-up – persons died), ^2^Of the three persons without 6 weeks follow-up visits, one person did Snot have a 6 months follow-up visit before 16 July 2018. Of the six persons without 6 months follow-up visits, one person did not have a 12 months follow-up visit before 16 July 2018.

Since 2012, the Energy for life after ColoRectal cancer (EnCoRe) study is an ongoing prospective cohort investigating post-treatment lifestyle and health-related outcomes in CRC survivors. TNM-staged CRC patients I-III were recruited at diagnosis from three participating Dutch hospitals. Participants were visited by trained dietitians during five repeated home visits; at diagnosis and at 6 weeks, 6 months, 12 months and 24 months post-treatment. For the current analysis, data collected up until July 2018 were used. Figure 1 shows a flow diagram describing recruitment and follow-up of participants in the study. The decrease in number of participants during the follow-up measurements was mainly because participants had not yet reached all post-treatment time points in July 2018.

Men and women aged minimum 18 years old and diagnosed with stage I-III CRC were eligible, while individuals with stage IV CRC and comorbidities obstructing successful study participation (e.g. Alzheimer’s disease) were excluded. The Medical Ethics Committee of the University Hospital Maastricht and Maastricht University approved the study (Netherlands Trial Register number NL6904)^(14)^. All participants provided written informed consent.

### Dietary intake

To obtain quantitative data on food and beverage consumption, participants filled out a structured dietary record on seven consecutive days at all post-treatment time points. In the dietary record, participants reported consumed meals, foods and beverages with details on brand names, portion sizes and preparation. Participants received detailed oral and written instructions on how to fill out the dietary record. Additionally, all completed dietary records were checked by the dietitians.

Daily dietary intake was calculated utilising food calculation software (Compl-eat) and based on the Dutch Food Composition database (NEVO-2011), using existing or specifically created dietary food groups in the software based on the 2018 WCRF/AICR dietary sub-recommendations^(10)^. In this article, we specifically focus on the recommendations about limiting unhealthy dietary habits including fast foods, red and processed meat, alcohol and sugar-sweetened drinks. Fast foods were defined as UPF and classified according to the NOVA system that classifies food groups based on the extent of processing^(12,15)^. The UPF list from the NOVA classification was adapted by the National Cancer Institute to be in line with the WCRF/AICR report (i.e. food products made from white flour), national guidelines and to ensure that the adapted UPF group did not include food already accounted for in other dietary recommendations (i.e. sugar-sweetened drinks and processed meat). Consumption of fast foods was calculated by using energy percent of UPF consumed relative to total energy intake (EN%). Besides fast foods defined as UPF (EN%), we also looked at energy density of total food intake (kcal/100 g) used in previous operationalisations^(12,16)^. Red meat consumption (g/d) was based on reported intake of any kind of fresh raw meat that still needed to be prepared before consumption. Intake of processed meat (g/d) included reported intake of any meat that had been preserved and was ready for consumption. Calculation of alcohol intake was based on the nutrient value (ethanol) from the food calculation table for total ethanol (g/d) according to the reported dietary alcohol intake. Sugar-sweetened drinks (g/d) were calculated as the amount of sugar-containing drinks used (fruit drinks included). Details about included food products in each recommendation and additional information regarding methods and procedures applied for the assessment and coding of dietary records are extensively explained in Kenkhuis *et al.* ^(12)^

### Health-related quality of life, fatigue and chemotherapy-induced peripheral neuropathy

HRQoL outcomes including global quality of life, physical, role and social functioning and fatigue were measured with the well-validated cancer-specific European Organization for the Research and Treatment of Cancer Quality of Life Questionnaire-Core 30 (EORTC QLQ-C30)^(17,18)^. All scale scores were linearly transformed to a 0–100 scale, with higher scores on the functioning scales and global QoL reflecting better functioning or HRQoL, whereas higher symptom scale scores indicate more symptoms (i.e. worse fatigue). In addition, a summary score (SumSc) was calculated from the mean of 13 of the 15 subscores (excluding the financial difficulties and global QoL questions) according to recommendations by the EORTC Quality of Life Group^(18,19)^.

Besides the fatigue symptom scale from the EORTC QLQ-C30, fatigue was also assessed by the validated twenty-item Checklist Individual Strength (CIS) to enable a more comprehensive multidimensional assessment of fatigue^(20,21)^. The CIS consists of four subscales: subjective fatigue (range: 8–56), concentration problems (5–35), reduced motivation (4–28) and activity-related fatigue (3–21). In addition, a total fatigue score was derived by the summation of all subscales (range: 20–140). We included subjective fatigue, activity-related fatigue and the total fatigue score in the present analyses, since we expected dietary intake to be associated with the physical and subjective dimensions of fatigue and not the dimensions reflecting concentration problems and motivation. Higher scores indicate worse fatigue on all scales.

CIPN symptoms were measured with the EORTC QLQ-CIPN20. This twenty-item questionnaire consists of sensory, motor and autonomic subscales and a summary score^(22)^. All scale scores were linearly converted to a 0–100 scale^(23)^, with higher scores indicating more CIPN symptoms.

### Lifestyle, clinical and socio-demographic factors

Socio-demographic characteristics including age and sex and clinical information (i.e. cancer stage, chemotherapy/radiotherapy and tumour site) were retrieved from medical records. Self-reported data were collected on other factors, including educational level, current smoking status, presence of stoma and comorbidities at all time points^(24)^. BMI was calculated based on weight (kg) and height (m^2^) assessed by trained dietitians at every time point. Moderate-to-vigorous physical activity was calculated^(25)^ by adding up time spent on commuting, household, work and leisure time activities in the past week, as assessed by the Short QUestionnaire to ASsess Health-enhancing physical activity (SQUASH). Sedentary time was objectively assessed with the validated tri-axial MOX activity meter (Maastricht Instruments B.V.), as described previously by van Roekel *et al.* ^(26)^ Habitual dietary intake in the year prior to the diagnosis was assessed with a 253-item semi-quantitative FFQ at diagnosis^(27)^. To assess amounts of food intake, we combined frequencies of intake with standard portion sizes and household measures. Self-reported dietary intake data from the FFQ were converted into alcohol based on the NEVO-2011^(28)^.

### Statistical analyses

To describe the study population, we used descriptive analyses of socio-demographic, lifestyle and clinical variables at 6 weeks. To describe changes from 6 weeks up to 24 months post-treatment in dietary intake of fast foods, red and processed meat, alcohol and sugar-sweetened drinks, descriptive analyses were performed, overall and by sex.

Confounder-adjusted linear mixed models were used to assess longitudinal associations of fast foods, energy density of total food intake, red and processed meat, alcohol and sugar-sweetened drinks in relation to HRQoL, fatigue and CIPN outcomes between 6 weeks and 24 months post-treatment. The individual dietary recommendations were modelled continuously with units based on recommended portions per day and on relevant differences in portion sizes (i.e. per 5 energy percent UPF; 100 kcal/100 g/d total energy density; 100 g/d red meat; 50 g/d processed meat; 10 g/d alcohol and 250 g/d sugar-sweetened drinks). We adjusted regression models for an *a priori*-defined confounder set which included fixed (time-invariant) confounders including age at enrolment (years), sex and chemotherapy (yes, no) and time-variant confounders (measured at all post-treatment time points) like BMI (kg/m^2^), number of comorbidities (0, 1 and ≥ 2), moderate-to-vigorous physical activity (min/week), sedentary behaviour (h/d), total energy intake (kcal/d), stoma (yes/no) and time since diagnosis (months). We further applied the 10 % change-in-estimate method^(29)^ for assessing an additional set of potential confounders including education level (low, medium and high), radiotherapy (yes, no) and smoking (current, former and never); none of the variables led to > 10 % change in beta estimates of the dietary recommendations and were, therefore, not included in the main model. In addition, correlations between the dietary factor were generally weak (between –0·31 and 0·25), and these factors were hence not included as covariates. The use of random slopes was tested with a likelihood-ratio test; random slopes were added when the model improved statistically significantly. CIPN outcomes were only analysed for the subgroup of patients who received chemotherapy^(30)^. Inter- and intra-individual associations were disaggregated by adding centred person-mean values to the model to estimate inter-individual associations (i.e. average differences between participants over time) and individual deviations from the person-mean value to estimate intra-individual associations (i.e. within-participant changes over time)^(31)^. In addition, we performed a *post hoc* sensitivity analysis for UPF and processed meat with CIPN by analysing the association in non-chemotherapy participants as well.

To gain more insight into the observed results for alcohol consumption, we performed four *post hoc* analyses on alcohol. First, alcohol consumption was modelled as a categorical variable: no alcohol consumption; moderate alcohol consumption (1–14 glasses per week, reference category) and heavy alcohol consumption (> 14 glasses per week). Second, the association was assessed in only participants who reported to drink alcohol (at one or more time points), excluding alcohol abstainers at all time points. Third, the association was assessed with additional adjustment for habitual alcohol consumption assessed at diagnosis. Fourth, a time-lag analysis was performed to obtain insight into the possible direction of the longitudinal associations. In this analysis, alcohol at earlier time points was coupled with HRQoL and fatigue variables at subsequent time points to simulate a more natural direction of association. An inverse time-lag analysis coupling HRQoL and fatigue variables at earlier time points with alcohol at subsequent time points was also performed to simulate whether HRQoL and fatigue could influence alcohol intake.

Statistical analyses were performed using Stata 15.0 (StataCorp. 2017) with statistical significance set at *P* < 0·05 (two-sided).

## Results

Characteristics of included study participants (*n* 396) at 6 weeks post treatment are presented in Table 1. The mean age of participants was 67 years (sd: 9·1), 68 % were males and 51 % reported 2 or more comorbid conditions. Based on BMI, 30 % of participants had a BMI below 25·0 of which two participants were underweight, 44 % were overweight and 26 % were obese. A total of 320 (82 %) survivors adhered to the physical activity recommendation (≥ 150 min/week of moderate-to-vigorous physical activity) and 9 % were current smokers. With regards to treatment, 25 % received radiotherapy, of which the majority received pre-operative radiotherapy and only 2 participants received post-operative radiotherapy. In total, 18 % of the participants received pre-operative chemotherapy and 29 % received post-operative chemotherapy. In total, 39 % of the participants received chemotherapy because some participants received both pre- and post-operative chemotherapy. Pre-operative chemotherapy consisted of Capacetabine monotherapy, and in the case of post-operative chemotherapy, the majority (86 %) received Capacetabine + Oxaliplatin (CAPOX), 9 % received Capacetabine monotherapy and one person received 5FU + oxaliplatin (FOLFOX).

**Table 1.** Demographic, lifestyle and clinical characteristics of colorectal cancer survivors at 6 weeks post-treatment (Numbers and percentage)

	Total (*n* 396)*		Men (*n* 270)*		Women (*n* 126)*	
	*n*	%	*n*	%	*n*	%
Age (years)						
Mean	67·0		66·7		67·7	
sd	9·1		8·7		9·8	
Education†						
Low	107	27·1	58	21·6	49	38·9
Medium	149	37·7	102	37·9	47	37·3
High	139	35·2	109	40·5	30	23·8
Comorbidities‡						
0 comorbidities	91	23·0	71	26·3	20	16·0
1 comorbidity	102	25·8	78	28·9	24	19·2
≥ 2 comorbidities	202	51·1	121	44·8	81	64·8
Tumour stage						
Stage I	124	31·3	84	31·1	40	31·8
Stage II	100	25·3	66	24·4	34	27·0
Stage III	172	43·4	120	44·4	52	41·3
Cancer type						
Colon	250	63·1	162	60·0	88	69·8
Rectosigmoid and rectum	146	36·9	108	40·0	38	30·2
Treatment						
Chemotherapy (yes)	155	39·1	105	38·9	50	39·7
Radiotherapy (yes)	101	25·5	75	27·8	26	20·6
Stoma (yes)§	110	28·4	85	32·1	25	20·3
Lifestyle						
Smoking||						
Never	118	30·5	61	23·1	57	46·3
Former	235	60·7	177	67·1	58	47·2
Current	34	8·8	26	9·9	8	6·5
	Mean	sd	Mean	sd	Mean	sd
BMI, kg/m^2^ ‡	27·8	4·6	27·8	4·3	27·8	5·1
Underweight: < 18·5	2	0·5	0	0·0	2	1·6
Healthy weight: 18·5–24·9	117	29·6	82	30·4	35	28·0
Overweight: 25–29·9	173	43·8	117	43·3	56	44·8
Obese: ≥ 30	103	26·1	71	26·3	32	25·6
Moderate-to-vigorous physical activity, min/week¶						
Median	420		550		300	
IQR	645		750		340	
	*n*	%	*n*	%	*n*	%
Adherence to physical activity recommendation (yes)¶	320	82·0	232	86·9	88	71·5
Prolonged sedentary time. h/week**						
Mean	5·3		5·2		5·5	
sd	2·7		2·6		2·8	
Dietary intake††						
Ultra-processed foods (EN%)	35·5	10·6	34·9	10·4	36·6	11·1
Energy density (kcal/100 g)	172·6	28·1	176·4	27·8	164·6	27·3
Sugar-sweetened drinks (g/d)	133·6	158·1	143·0	162·2	113·6	147·7
Alcohol intake (g/d)	13·1	19·1	16·7	21·6	5·5	8·3
Red meat intake (g/w)	592·4	294·6	648·2	302·8	473·6	236·8
Processed meat intake (g/w)	325·2	209·7	365·5	221·8	239·5	149·0

d, day; EN%, energy percentage; g, grams; h, hours; min, minutes; sd, standard deviation; w, week; IQR, interquartile range.

* Percentages may not add to 100 due to rounding.

† Data missing for one participants (male).

‡ Data missing for one participant (female).

§ Data missing for eight participants (five males and three females).

|| Data missing for nine participants (six males and three females).

¶ Data missing for six participants (3 males and three females).

** Sedentary behaviour available for 325 participants (220 males and 105 females).

†† Dietary intake available for 383 participants (261 males and 122 females).

### Changes in dietary intake up to 24 months post-treatment

**Fig. 2.**
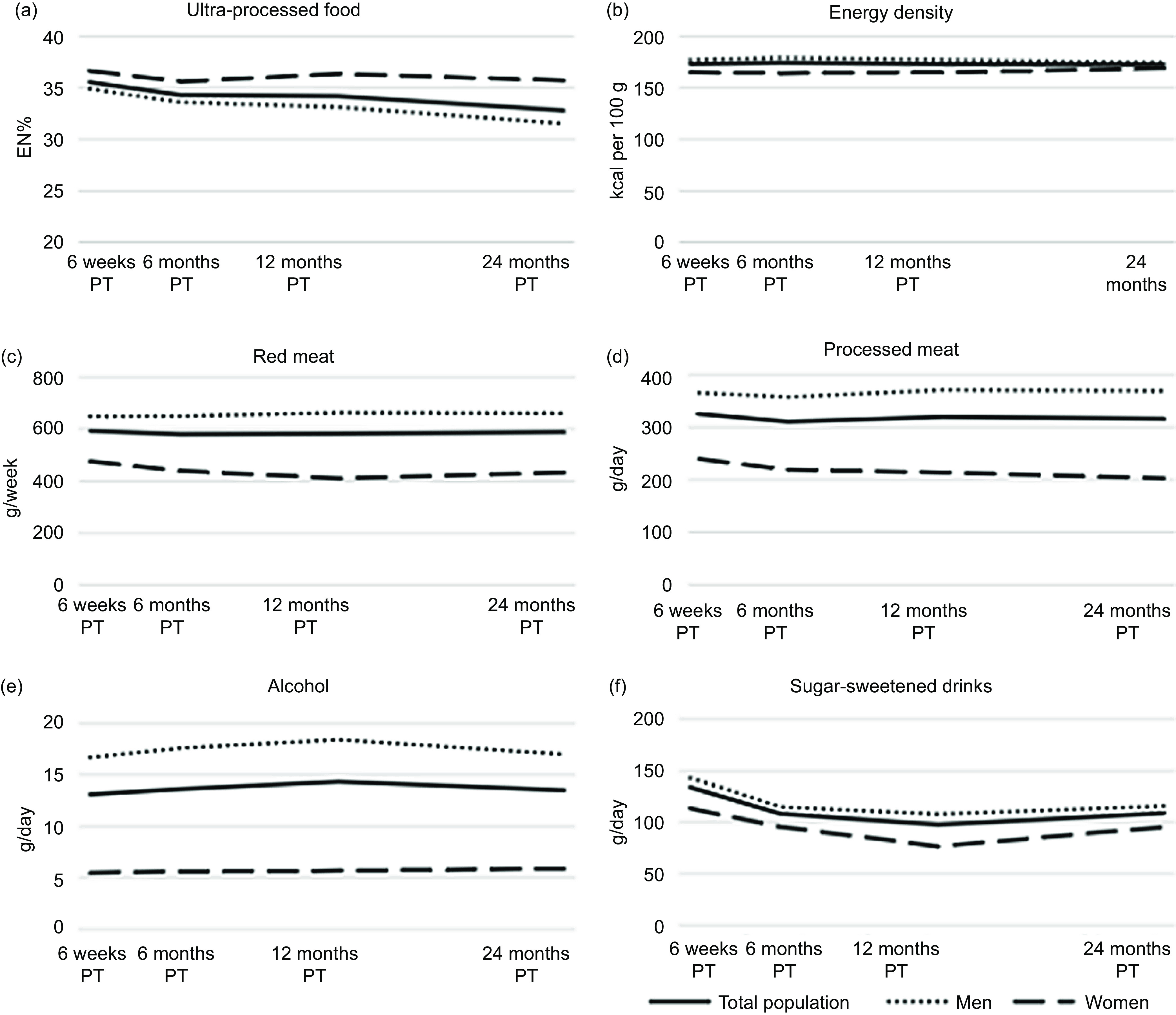
Course of ultra-processed food (a), energy density (b), red meat (c), processed meat (d), alcohol intake (e), and sugar-sweetened intake (f) and from 6 weeks up to 24 months post-treatment (PT) in stage I to III colorectal cancer survivors in the EnCoRe study.

A slight decline over time was seen for UPF intake (Fig. 2(a)), which was larger in men than in women. At 6 weeks, average daily energy percentage for UPF was 36 EN%, which declined to 33 EN% at 24 months. No decline was seen for energy-dense foods (Fig. 2(b)). Average daily consumption of energy-dense foods remained around 172 kcal/100 g at all time points. Sugar-sweetened drinks declined from mean intake of 134 g/d at 6 weeks to 109 g/d at 24 months (Fig. 2(c)). At 6 weeks, 32 % (*n* 122) adhered to the recommendation to not consume alcohol at all. On average, men drank triple the amount of alcohol compared with women and a slight mean increase in consumption was observed at 12 months for men (Fig. 2(d)). At 6 weeks, 20 % (*n* 77) and 2 % (*n* 6) adhered to the recommendation regarding red and processed meat consumption with an average consumption of 648 g/w and 366 g/w at 6 weeks, respectively for men and 474 g/w and 240 g/w, respectively for women (Fig. 2(e) and (f)).

HRQoL increased, whereas fatigue and CIPN symptoms decreased from 6 weeks up to 24 months post-treatment. The changes over time for HRQoL, fatigue and CIPN are explained in detail in Kenkhuis *et al.* ^(32)^

### Longitudinal associations of dietary intake with health-related quality of life and fatigue

**Fig. 3.**
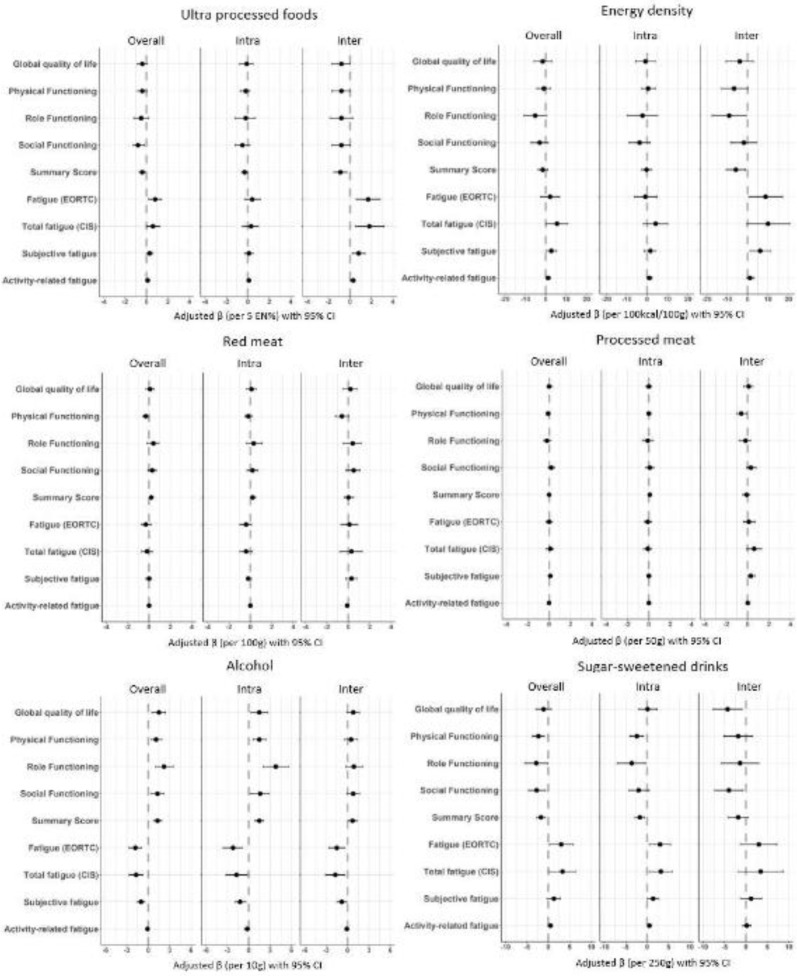
Forest plots showing the confounder-adjusted beta’s (*β*) and 95 % CI for the overall longitudinal, intra and inter-individual associations of ultra-processed food (UPF), energy density, red and processed meat, alcohol and sugar-sweetened drinks in relation to health-related quality of life and fatigue in stage I-III colorectal cancer survivors followed-up from 6 weeks up to 24 months post-treatment. The confounder-adjusted betas are modelled based on recommended portions per day and on relevant differences in portion sizes (i.e. per 5 energy percent per day UPF; 100 kcal/100 g energy density; 100 g per day red meat; 50 g per day processed meat; 10 g per day alcohol and 250 g per day sugar-sweetened drinks). The x-axis scale differs per food group.

In confounder-adjusted models assessing overall longitudinal associations from 6 weeks to 24 months post-CRC treatment (Fig. 3 and online Supplementary Table 1), higher energy percentage of UPF was associated with lower social functioning (*β* per 5 EN%: –0·8; 95 % CI: –1·3, –0·2) and with higher levels of fatigue (EORTC: 0·8; 0·2, 1·4). Increased energy density of total food intake was associated with higher levels of total fatigue (CIS: *β* per 100 kcal/100 g: 5·5; 0·2, 10·8) and higher levels of subjective fatigue (2·7; 0·1, 5·3). Separate models testing inter- and intra-individual associations (Fig. 3 and online online Supplementary Table 1) showed that the associations for UPF and energy density were mostly driven by the inter-individual component. A difference on average in UPF intake of 5 EN% between individuals over time was predominantly associated with higher levels of fatigue (EORTC: 1·7; 0·6, 2·8), total fatigue (CIS: 1·8; 0·5, 3·2), subjective fatigue (0·8; 0·2, 1·4) and activity-related fatigue (0·3; 0·0, 0·5).

A higher intake of sugar-sweetened drinks was associated with lower levels on all functioning scales and with higher levels of fatigue. This association appeared to be mostly driven by the intra-individual component, indicating that an increase in sugar-sweetened drinks over time within individuals, and not a difference in average consumption of sugar-sweetened drinks between individuals, was predominantly associated with worse HRQoL and functioning outcomes and more fatigue over time. In particular, intra-individual analyses showed that an increase of 250 g/d in sugar-sweetened drinks within individuals over time was statistically significantly associated with lower physical functioning (–2·4; −4·1, –0·8), role functioning (–3·6; −7·0, –0·3), higher levels of fatigue (EORTC: 3·0; 0·5, 5·5), total fatigue (CIS: 3·2; 0·5, 5·9) and subjective fatigue (1·4; 0·0, 2·7).

A higher intake of alcohol was associated with higher HRQoL and less fatigue. This association appeared to be mostly driven by the intra-individual component for global QoL and functioning and by both intra-individual changes as well as inter-individual differences for fatigue. An increase of 10 g/d in alcohol within individuals over time was statistically significantly associated with higher global QoL (1·5; 0·3, 2·7), physical functioning (1·5; 0·6, 2·4), role functioning (3·8; 2·0, 5·6) and social functioning (1·6; 0·2, 2·9) and with lower levels of fatigue (EORTC: −2·2; –3·6, –0·9), total fatigue (CIS: −1·7; −3·2, –0·2) and subjective fatigue (–1·2; −1·9, –0·4).

No statistically significant associations were found for red meat and processed meat with HRQoL and fatigue outcomes (Fig. 3 and online Supplementary Table 1).

### Longitudinal associations of dietary intake with chemotherapy-induced peripheral neuropathy

**Fig. 4.**
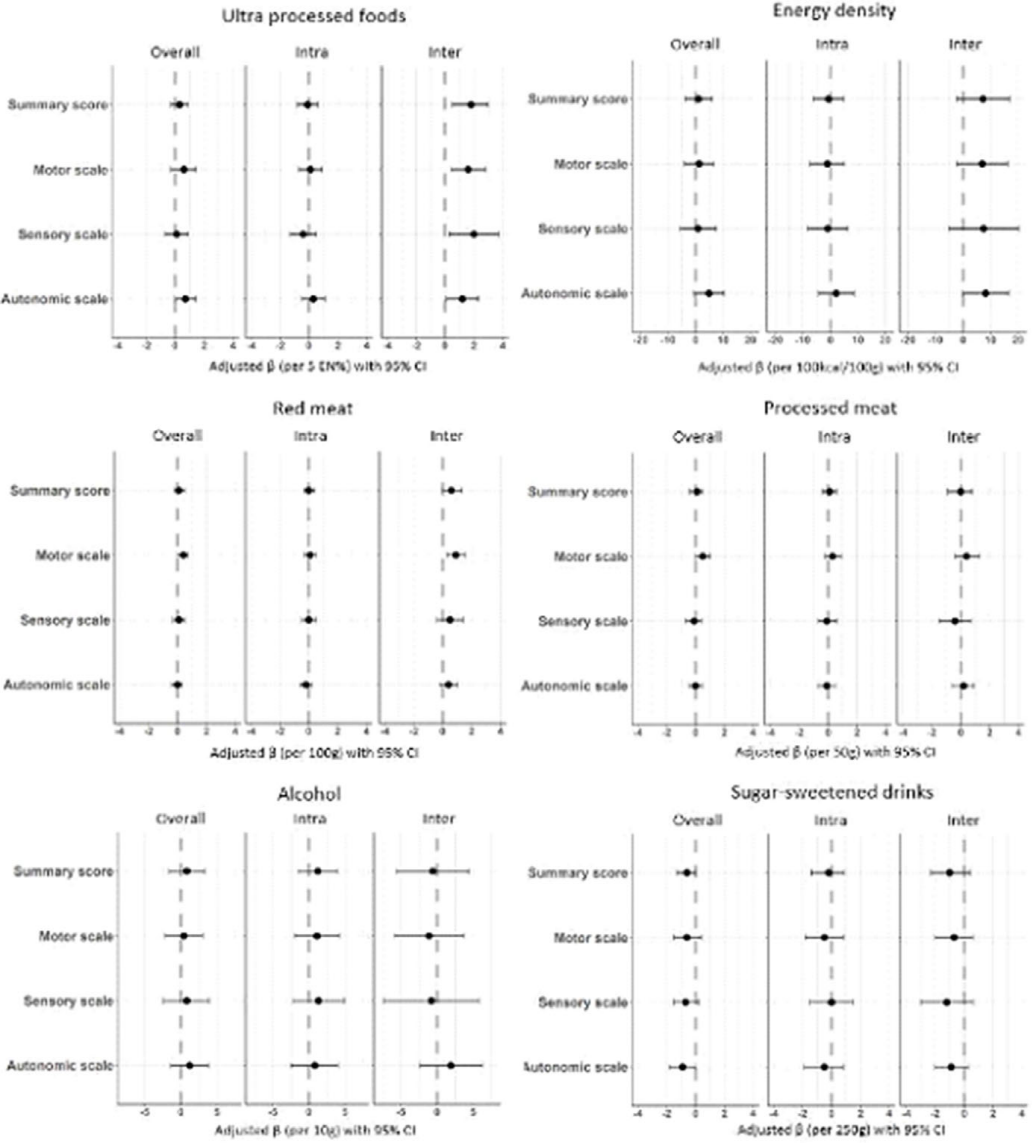
Forest plots showing the confounder-adjusted beta’s (*β*) and 95 % CI for the overall longitudinal, intra and inter-individual associations of ultra-processed food (UPF), energy density, red and processed meat, alcohol and sugar-sweetened drinks in relation to chemotherapy-induced peripheral neuropathy (CIPN) in stage I-III colorectal cancer survivors followed-up from 6 weeks up to 24 months post-treatment. The confounder-adjusted betas are modelled based on recommended portions per day and on relevant differences in portion sizes (i.e. per 5 energy percent per day UPF; 100 kcal/100 g energy density; 100 g per day red meat; 50 g per day processed meat; 10 g per day alcohol and 250 g per day sugar-sweetened drinks). The x-axis scale differs per food group.

For CIPN outcomes (Fig. 4 and online Supplementary Table 2), an inter-individual association was found for higher UPF and processed meat intake with more CIPN symptoms. Individuals who had on average 5 EN% more daily intake of UPF over time reported higher scores on the CIPN motoric (1·6; 0·4, 2·8), sensoric (2·0; 0·3, 3·7) and autonomic (1·2; 0·1, 2·3) subscales in comparison with individuals with lower UPF intakes, and individuals who consumed on average 50 g per day more of processed meat over time reported higher scores on the CIPN motoric subscale (0·9; 0·3,1·5). *Post hoc* sensitivity analyses showed no statistically significant inter-individual associations between UPF and CIPN in participants who did not receive chemotherapy, but the inter-individual association between processed meat and CIPN motor subscale observed in participants who received chemotherapy remained in participants who did not receive chemotherapy (data not shown).

### Additional *post hoc* analyses for alcohol consumption

When alcohol was analysed as a categorical variable, participants who did not drink alcohol had statistically significantly lower levels on all functioning subscales of the EORTC QLQ-C30 and higher levels of fatigue according to the EORTC QLQ-C30 and CIS compared with participants reporting moderate alcohol consumption (Table 2). Moreover, heavy drinkers (> 14 glasses per week) reported statistically significantly higher levels of physical functioning and lower levels of total fatigue (CIS) and subjective fatigue over time compared with participants reporting moderate alcohol consumption. Analysis in only participants who consumed alcohol, i.e., excluding participants who never drank alcohol at any of the follow-up time points (abstainers), showed no attenuation or differences compared with the results of the main analysis. When adjusting for habitual alcohol intake assessed at diagnosis, the overall associations of alcohol consumption with HRQoL and fatigue did not attenuate and were even slightly stronger compared with results of the main analysis (Table 2 *v*. online Supplementary Table 1). In comparison with results of the main analysis, the overall associations of alcohol consumption with HRQoL and fatigue were attenuated within time-lag analysis (e.g., physical functioning: *β* per 10 g/d: 0·5; 95 % CI: –0·3, 1·3; Table 2). In time-lag analysis, estimates of inter-individual associations were mostly statistically significant and slightly bigger compared with the main analyses (online Supplementary Table 1), while the intra-individual associations were not statistically significant anymore, being slightly smaller or even tended to be reversed for some outcomes (e.g. physical functioning: *β* per 10 g/d: –0·5; 95 % CI: –1·6, 0·5). In the inverse time-lag analysis, higher physical functioning was associated with increased consumption of alcohol overall and driven by the within component (*β* per 10 points: 0·9:0·1, 1·6), whereas more fatigue was associated with decreased consumption of alcohol only between individuals (e.g. EORTC fatigue: *β* per 10 points: –1·5: –2·6, –0·5).

**Table 2.** Additional post-hoc analyses for alcohol (categorical, with additional adjustment and time-lag analysis) in relation to health-related quality of life and fatigue (Coefficient values and 95 % confidence intervals)

		EORTC QLQ-C30												CIS					
		Global QoL (0–100)		Physical functioning (0–100)		Role functioning (0–100)		Social functioning (0–100)		SumSc (0–100)		Fatigue (0–100)		Fatigue (20–140)		Subjective fatigue (8–56)		Activity fatigue (3–21)	
		*β*	95 % CI	*β*	95 % CI	*β*	95 % CI	*β*	95 % CI	*β*	95 % CI	*β*	95 % CI	*β*	95 % CI	*β*	95 % CI	*β*	95 % CI
Categorical analysis†																			
No alcohol consumption		–2·8*	–5·3, –0·3	–3·7*	–5·7, –1·7	–5·9*	–9·4, –2·3	–3·9*	–6·5, –1·2	–3·6*	–5·0, –2·2	5·5*	2·6, 8·5	4·5*	1·2, 7·8	2·0*	0·4, 3·6	0·9	0·2, 1·7
Moderate alcohol consumption‡		REF		REF		REF		REF		REF		REF		REF		REF		REF	
Heavy alcohol consumption‡		1·4	–1·2, 4·0	2·1*	0·0, 4·2	3·1	–0·7, 6·8	2·6	–0·1, 5·4	1·3	–0·2, –2·2	–2·0	–5·1, 1·1	–3·8*	–7·3, –0·3	–2·1*	–3·8, –0·4	–0·6	–1·4, 0·3
Additional adjustment for habitual alcohol intake																			
Alcohol (10g/d)	Adj.	1·5*	0·5, 2·5	1·4*	0·7, 2·2	2·7*	1·4, 4·1	1·5*	0·5, 2·5	1·4*	0·8, 1·9	–2·2*	–3·3, –1·0)	–2·2*	–3·5, –0·9	–1·3*	–1·9, –0·7	–0·3*	–0·6, –0·0
	Within	1·5*	0·3, 2·7	1·5*	0·6, 2·4	3·8*	2·0, 5·7	1·6*	0·3, 2·9	1·4*	0·8, 2·1	–2·2*	–3·6, –0·9	–1·7*	–3·2, –0·2	–1·1*	–1·9, –0·4	–0·2	–0·5, 0·1
	Between	1·5	–0·1, 3·2	1·4	–0·2, 3·0	1·3	–0·8, 3·4	1·4	–0·2, 3·0	1·2*	0·0, 2·3	–2·0	–4·1, 0·0	–3·6*	–6·2, 1·1	–1·8*	–3·0, –0·6	–0·5	–0·9, 0·0
Time lag analysis	Unadj	1·0*	0·3, 1·8	0·5	–0·1, 1·2	1·4*	0·5, 2·5	0·6	–0·1, 1·4	0·5	–0·0, 0·9	–1·4*	–2·3, –0·5	–0·6	–1·7, 0·5	–0·3	–0·8, 0·2	–0·0	–0·2, 0·2
Alcohol (10g/d)	Adj.‡,§	0·9*	0·2, 1·6	0·5	–0·3, 1·3	1·3*	0·2, 2·5	1·3*	0·4, 2·3	0·4*	0·1, 0·8	–1·4*	–2·4, –0·4	–0·8	–2·0, 0·4	–0·3	–0·9, 0·2	0·0	–0·2, 0·3
	Within	0·4	–1·1, 2·0	–0·5	–1·6, 0·5	–0·4	–2·6, 1·7	–0·7	–2·3, 0·8	–0·5	–1·3, 0·3	–0·3	–2·1, 1·5	1·4	–0·5, 3·4	0·7	–0·3, 1·6	0·3	–0·1, 0·8
	Between	1·3*	0·4, 2·3	0·7	–0·2, 1·7	1·2*	0·0, 2·4	1·3*	0·4, 2·2	0·9*	0·2, 1·5	–1·8*	–3·0, –0·7	–2·1*	–3·5, –0·6	–0·9*	–1·6, –0·2	–0·1	–0·4, 0·2

EORTC QLQ-C30, European Organization for the Research and Treatment of Cancer Quality of Life; *β*, beta-coefficient; QoL, quality of life; g/d, gram per day.

* Indicates a statistically significant association.

† Alcoholic consumption was modelled as a categorical variable: no alcohol consumption; moderate alcohol consumption: 1–14 glasses per week (reference category); and heavy alcohol consumption: > 14 glasses per week for both men and women.

‡ Model adjusted for sex (male/female), age enrolment (years), co-morbidities (0, 1 and ≥ 2), weeks since end of treatment (weeks), chemotherapy (yes/no), BMI (kg/m^2^), moderate-to-vigorous physical activity (min/week), sedentary time (h/d), energy intake (kcal/d) and stoma (yes/no).

§ The beta-coefficients represent the overall longitudinal difference in the outcome score.

## Discussion

Within this longitudinal study of stage I-III CRC survivors, we studied relations of post-treatment intake of UPF, energy density of total food intake, red and processed meat, alcohol and sugar-sweetened drinks with HRQoL, fatigue and CIPN. Mean dietary intake in the period from 6 weeks until 24 months after the end of CRC treatment remained relatively stable over time for energy density and red and processed meat. Mean intakes of UPF and sugar-sweetened drinks decreased over time, and mean alcohol intake slightly increased at 12 months. In confounder-adjusted analyses, we observed that higher intakes of UPF, energy density of total food intake and sugar-sweetened drinks were associated with lower HRQoL and higher levels of fatigue. In contrast, higher alcohol consumption was associated with higher HRQoL and lower levels of fatigue, although these associations attenuated within the time-lag analysis. Lastly, in CRC patients who had received chemotherapy, higher UPF intake and processed meat were associated with increased CIPN symptoms up to 24 months post-treatment.

To the best of our knowledge, this is the first study that assessed longitudinal relationships of fast foods, red and processed meat, alcohol and sugar-sweetened drinks with HRQoL, fatigue and CIPN in CRC survivors, from 6 weeks up to 24 months post-treatment. Fast foods operationalised as UPF and energy density followed different trends in time, suggesting that something slightly different was measured by these variables. Associations for UPF and energy density appeared to be mainly driven by between-person differences, indicating that individuals with higher UPF intake reported on average more fatigue over time than individuals with lower intakes. The inter-individual associations for UPF and energy density of total food intake suggest a difference in overall dietary pattern between CRC survivors. Richardson demonstrated that biological, psychological, social and personal factors influence the onset, impact, expression, duration, pattern and severity of the fatigue experience^(33)^. A healthy lifestyle, including a healthy diet, often co-exists with, for instance, better sleep and a larger social network^(34)^. Moreover in CRC patients who had received chemotherapy, individuals with higher UPF and processed meat intake reported on average increased CIPN symptoms on all subscales. In individuals who did not receive chemotherapy, no association was found between higher UPF and CIPN, whereas the association between higher processed meat intake and CIPN remained. This may indicate that an interaction between UPF and chemotherapy has occurred, whereas this interaction did occur between processed meat and chemotherapy. Processed meat might thus not specifically be associated with CIPN, which is related to chemotherapy, but may resemble CIPN like symptoms. Among women with breast cancer, it was found that citrus fruit and grain consumption may play a role in neuropathy experience of some women undergoing chemotherapy^(35)^. Refined grains, part of our UPF list, tend to have high glycaemic load and are associated with increased risk of diabetes and complications, including peripheral neuropathy^(36)^. Another possible explanation could be that eating foods that trigger inflammation could increase pain symptoms associated with neuropathy by accelerating the inflammatory process^(37)^. These foods can vary from person to person, but most commonly include fried foods, margarine and refined grains^(37)^, all part of our UPF list.

Somewhat unexpectedly, post-treatment increases in alcohol intake within individuals were associated with better HRQoL and less fatigue. For alcoholic consumption, time-lag analysis showed attenuated or even reversed intra-individual associations and slightly increased inter-individual associations. The inverse time-lag analysis also showed associations for higher physical functioning and less fatigue with increased alcohol consumption. This suggests a bidirectional relation between alcohol and HRQoL and fatigue outcomes. Participants who start feeling better and less fatigued in the post-treatment period may be more likely to consume alcohol in comparison with people that do not feel better yet. This also suggests that it does not appear to be a biological effect of alcohol on HRQoL or fatigue. Instead, alcohol seems to be associated with a general feeling of well-being, and our findings may be a reflection of a return back to normal (social) life after the end of cancer treatment^(38–40)^.

We observed that higher intakes of sugar-sweetened drinks were associated with lower HRQoL and higher levels of fatigue, driven by both intra-individual changes and inter-individual differences over time. Importantly, we observed that the intake of sugar-sweetened drinks decreased on average in the period from 6 weeks up to 24 months after the end of treatment. Consequently, this average decrease implies a positive influence on HRQoL and fatigue. In other words, the average decrease in sugar-sweetened drink consumption in the first 24 months post-treatment appears to reflect a beneficial change that was already adopted by our CRC survivor population. A similar decrease was also seen in another CRC survivor population^(41)^.

In terms of clinical relevance, the observed effect sizes from the fully adjusted models were smaller than the minimal clinically important differences^(42–44)^. However, these associations may become larger when adhering to more recommendations, as recommended by WCRF/AICR, who consider their lifestyle recommendations as a package directing people towards a healthy lifestyle and having the largest impact when taken together. Even small changes could already be beneficial, as all small changes accumulate when individuals adhere more or to more recommendations.

### Strengths and limitations

An important strength of the current study is the prospective nature and repeated-measures design. In addition, a major strength of this study was the availability of extensive 7-d food diary measurements of dietary intake, which enabled quantitative assessment of UPF and energy density, sugar-sweetened drinks, alcohol consumption and red and processed meat intake, consequently being more accurate than commonly used FFQ data. Additionally, assessment of dietary intake on multiple days through food diaries makes the information gathered more reliable^(45)^. Other strengths of our study included the high response rates during follow-up (> 90 %), the limited number of missing data resulting from intensive data collection methods and the availability of extensive data on potential confounders. Furthermore, the mixed models enabled disentangling of inter- and intra-individual associations and we added an additional time-lag model, thereby providing valuable insights into the nature of the longitudinal associations despite the limitation in terms of power.

There are also limitations that should be considered. Based on these observational data, and although time-lag analyses provide some insight, we cannot be sure of the direction of associations between dietary intake and HRQoL, fatigue and CIPN. Intervention studies will be necessary to infer causality. In addition, the limited response rate at diagnosis (45 %) might have resulted in a selection bias. Participants with less favourable dietary conditions and lower HRQoL may have been less likely to participate and this may have led to an attenuation of associations. Moreover, because we had no information on HRQoL and fatigue at diagnosis, as well as complete information about possible recurrences during post-treatment follow-up, we were not able to adjust for these potential confounders. Finally, we cannot rule out the possibility of false-positive findings due to the large number of tests performed.

### Conclusion

In conclusion, we showed that increased post-treatment intake of UPF, energy-dense foods and sugar-sweetened drinks are longitudinally associated with worsened HRQoL and more fatigue in CRC survivors, while higher intake of UPF and processed meat was associated with increased CIPN symptoms. In contrast, increased post-treatment alcohol intake was longitudinally associated with better HRQoL and less fatigue. This finding likely reflected that participants who started to feel better in the period of recovery after cancer treatment also increased their alcohol use. This research can ultimately contribute to more specific dietary guidelines, in particular limiting fast foods and sugar-sweetened drink consumption might be of interest for CRC survivors in order to improve their HRQoL and symptoms in the years after treatment.

## Supplementary material

For supplementary material accompanying this paper visit https://doi.org/10.1017/S0007114522003051. click here to view supplementary material
