# Supplementary material for: Longitudinal associations of fast foods, red and processed meat, alcohol and sugar-sweetened drinks with quality of life and symptoms in colorectal cancer survivors up to 24 months post-treatment
Source: Br J Nutr. 2022 Sep 27;130(1):114–26. doi: 10.1017/S0007114522003051 (PMC10244013; doi:10.1017/S0007114522003051)
Supplement: Supplementary file 1 [file S0007114522003051sup001.docx]

Supplemental Table 2: Overall longitudinal, intra-individual and inter-individual associations between ultra-processed foods, energy density, sugar-sweetened drinks, alcohol, red and processed meat intake and chemotherapy-induced peripheral neuropathy in stage I to III colorectal cancer survivors from 6 weeks to 24 months post-treatment

|  |  | EORTC QLQ-CIPN20 | | | | | | | | |
| --- | --- | --- | --- | --- | --- | --- | --- | --- | --- | --- |
|  | SumScore | | Motoric | | Sensoric | | | Autonomic | |  |
|  | β (95% CI) | | β (95% CI) | | β (95% CI) | | | β (95% CI) | |  |
| Ultra-processed foods (5EN%) | Unadjusted | 0.2 | (-0.4,0.7) | 0.5 | (-0.1,1.1) | 0.0 | (-0.7,0.7) | 0.5 | | (-0.0,1.1) |
|  | Adjusted^abe^ | 0.3 | (-0.3,0.9) | 0.6 | (-0.3,1.4) | 0.1 | (-0.7,0.9) | 0.7 | | (-0.0,1.4) |
|  | Within^ac^ | -0.1 | (-0.8,0.6) | 0.1 | (-0.7,0.9) | -0.4 | (-1.3,0.5) | 0.3 | | (-0.5,1.1) |
|  | Between^ad^ | 1.8* | (0.5,3.0) | 1.6* | (0.4,2.8) | 2.0* | (0.3,3.7) | 1.2* | | (0.1,2.3) |
| Energy density (100kcal/100g) | Unadjusted | 0.4 | (-3.8,4.6) | 0.6 | (-4.2,5.3) | 0.8 | (-4.7,6.3) | 2.1 | | (-2.3,6.5) |
|  | Adjusted^abe^ | 1.1 | (-3.8,6.1) | 1.3 | (-4.1,6.7) | 0.9 | (-5.6,7.5) | 4.9 | | (-0.7,10.4) |
|  | Within^ac^ | -0.7 | (-6.3,4.8) | -1.1 | (7.4,5.2) | -1.0 | (-8.3,6.3) | 2.1 | | (-4.4,8.6) |
|  | Between^ad^ | 7.3 | (-2.3,16.9) | 7.0 | (2.3,16.2) | 7.5 | (5.2,20.2) | 8.2 | | (-0.2,16.6) |
| Red meat (100g/d) | Unadjusted | 0.0 | (-0.4,0.4) | 0.2 | (-0.2,0.6) | -0.1 | (-0.6,0.4) | -0.2 | | (-0.6,0.2) |
|  | Adjusted^abe^ | 0.1 | (-0.4,0.5) | 0.5* | (0.0,1.0) | -0.1 | (-0.7,0.4) | 0.0 | | (-0.5,0.5) |
|  | Within^ac^ | 0.1 | (-0.4,0.6) | 0.3 | (-0.2,0.9) | -0.1 | (-0.7,0.6) | -0.1 | | (-0.7,0.5) |
|  | Between^ad^ | 0.0 | (-0.9,0.8) | 0.4 | (-0.4,1.3) | -0.4 | (-1.5,0.7) | 0.2 | | (-0.6,0.9) |
| Processed meat (50g/d) | Unadjusted | 0.1 | (-0.2,0.4) | 0.3 | (-0.0,0.6) | 0.1 | (-0.3,0.4) | -0.1 | | (-0.4,0.2) |
|  | Adjusted^abe^ | 0.1 | (-0.2,0.5) | 0.4* | (0.0,0.7) | 0.1 | (-0.3,0.5) | 0.0 | | (-0.4,0.3) |
|  | Within^ac^ | 0.0 | (-0.3,0.4) | 0.1 | (-0.3,0.5) | 0.0 | (-0.5,0.5) | -0.2 | | (-0.6,0.2) |
|  | Between^ad^ | 0.6 | (-0.0,1.3) | 0.9* | (0.3,1.5) | 0.5 | (-0.4,1.4) | 0.4 | | (-0.2,1.0) |
| Sugar-sweetened drinks (250g/d) | Unadjusted | -0.7 | (-1.5,0.1) | -1.0* | (-1.8,-0.1) | -0.5 | (-1.5,0.5) | -0.8* | | (-1.5,-0.0) |
|  | Adjusted^abe^ | -0.6 | (-1.3,0.0) | -0.6 | (-1.5,0.4) | -0.7 | (-1.5,0.2) | -0.9 | | (-1.8,0.0) |
|  | Within^ac^ | -0.2 | (-1.4,0.9) | -0.5 | (-1.8,0.8) | 0.0 | (-1.5,1.5) | -0.5 | | (-1.9,0.8) |
|  | Between^ad^ | -1.0 | (-2.3,0.4) | -0.7 | (-2.0,0.6) | -1.2 | (-3.0,0.6) | -0.9 | | (-2.0,0.3) |
| Alcohol (10g/d) | Unadjusted | 1.1 | (-1.1,3.3) | 1.1 | (-1.4,3.6) | 0.7 | (-2.3,3.6) | 1.6 | | (-0.7,3.9) |
|  | Adjusted^abe^ | 0.8 | (-1.6,3.2) | 0.4 | (-2.2,3.0) | 0.8 | (-2.4,3.9) | 1.2 | | (-1.4,3.8) |
|  | Within^ac^ | 1.2 | (-1.5,3.9) | 1.1 | (-2.0,4.2) | 1.3 | (-2.3,4.9) | 0.8 | | (-2.5,4.0) |
|  | Between^ad^ | -0.6 | (-5.6,4.4) | -1.1 | (-5.9,3.7) | -0.8 | (-7.4,5.8) | 1.9 | | (-2.4,6.3) |

Abbreviations: EORTC QLQ, European Organization for the Research and Treatment of Cancer Quality of Life; CIPN, chemotherapy-induced peripheral neuropathy; β, beta-coefficient; CI, confidence interval; g/d, gram per day; EN%, energy percentage; g/w, gram per week.

^a^ Model adjusted for sex (male/female), age enrolment (years), co-morbidities (0, 1, ≥2), weeks since end of treatment (weeks), body mass index (kg/m^2^), moderate-to-vigorous physical activity (min/week), sedentary time (hours/day), energy intake (kcal/day) and stoma (yes/no).

^b^ The beta-coefficients represent the overall longitudinal difference in the outcome score.

^c^ The beta-coefficients represent the change in the outcome score over time within individuals.

^d^ The beta-coefficients represent the difference in the outcome score between individuals.

^e^ A random slope was added to the model for sugar-sweetened drinks with summary scale, sensory scale and autonomic scale; red meat with motoric scale; energy density with autonomic scale; ultra-processed food with motoric scale and autonomic scale; no random slope was added to the models for alcohol and processed meat intake (see Methods).

* Indicates a statistically significant association
